# A novel mutation of WFS1 gene in a Chinese patient with Wolfram syndrome: a case report

**DOI:** 10.1186/s12887-018-1091-1

**Published:** 2018-03-17

**Authors:** Min Li, Jia Liu, Huan Yi, Li Xu, Xiufeng Zhong, Fuhua Peng

**Affiliations:** 1 Multiple Sclerosis Center, Department of Neurology, the Third Affiliated Hospital of Sun Yat-Sen University, Guangzhou, Guangdong 510630 China; 2 State Key Laboratory of Ophthalmology, Zhongshan Ophthalmic Center, Sun Yat-sen Univeristy, Guangzhou, Guangdong 510060 China

**Keywords:** Wolfram syndrome, WFS1 gene, Mutation

## Abstract

**Background:**

Wolfram syndrome (WS), caused by mutations of the Wolfram syndrome 1 (WFS1) gene on chromosome 4p16.1, is an autosomal recessive disorder characterized by diabetes insipidus (DI), neuro-psychiatric disorders, hearing deficit, and urinary tract anomalies.

**Case presentation:**

Here we report a 11-year-old Chinese boy who presented with visual loss, was suspected with optic neuritis (ON) or neuromyelitis optica (NMO) and referred to our department for further diagnosis. Finally he was diagnosed with WS because of diabetes mellitus (DM) and optic atrophy (OA). Eight exons and flanking introns of WFS1 gene were analyzed by sequencing. A novel mutation c.1760G > A in WFS1 gene of exon 8 was identified.

**Conclusion:**

This report reviews a case of WS associated with a novel mutation, c.1760G > A in WFS1 gene of exon 8, and emphasizes that WS should be taken into account for juveniles with visual loss and diabetes mellitus.

## Background

Wolfram syndrome (WS) is a rare autosomal recessive disorder, mainly associated with juvenile-onset type 1 diabetes mellitus (T1DM) and optic atrophy (OA). Diabetes insipidus (DI), neuro-psychiatric disorders, hearing deficit, and urinary tract anomalies develop in many patients [1, 2].

Mutations of the WFS1 gene on chromosome 4p16.1 are in charge of the clinical manifestations in majority of patients with WS [3, 4]. WFS1 gene encodes wolframin, an 890-amino acid glycoprotein localized primarily in the endoplasmic reticulum (ER) [5]. Genetic analyses in WS have identified a wide spectrum of mutations, including mis-sense, frame shifting, nonsense, and splicing mutations, predominantly located in exon 8 (80–90%) [6].

Here we report a WS case onset with OA and T1DM in a Chinese juvenile. A novel mutation c.1760G > A in WFS1 gene of exon 8 was found, which was never reported before.

## Case presentation

A 11-year-old boy was admitted to our department with a 1-year history of progressive visual loss. He was initially taken as pseudomyopia without treatment. Four months ago, he was diagnosed with T1DM and found bilateral OA by brain magnetic resonance image (MRI), optic coherence temography (OCT) and visual evoked potential (VEP) (no pictures provided) in the local hospital. And he was treated with compound anisodine hydrobromide injection and mouse nerve factor injection for one and a half months without alleviating. Then the boy was referred to our department with suspicious of ON or NMO.

The patient was hospitalized in local hospital because of a sudden faint in school 4 months ago. Some of his laboratory tests were as follows: finger point blood glucose test > 33.3 mmol/L, blood glucose 38 mmol/L, HbAc 15.8%, and positive urine sugar and ketone. His mother recallded that he was polydipsia, polyphagia and polyuria with weight loss for 1 month. So he was diagnosed with ‘type 1 diabetes mellitus (DM1), ketoacidosis and coma’, and treated with insulin pump of Medtronic in the local hospital.

**Fig. 1:**
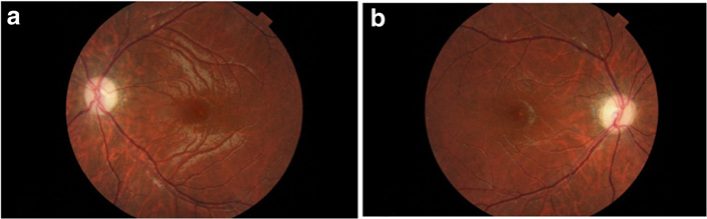
Photographic images of eyes. Fundus image discloses marked left (**a**) and right (**b**) atrophic optic discs with temporal pallor

**Fig. 2:**
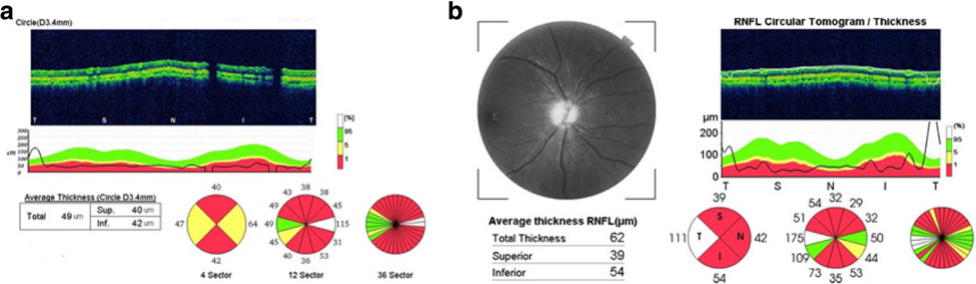
OCT. Measure around the disc shows thin retina in the left (**a**) and right (**b**) eyes

**Fig. 3:**
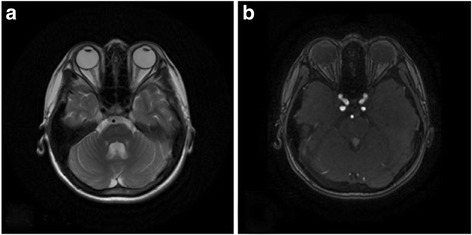
Cranium MRI. MRI shows bilateral optic nerve atrophy on T2WI (**a**) and T1W+C (**b**)

Some of his main investigations in our hospital revealed as follows. ANA+ENA + ANCA, TSH in serum, AQP4 and OCB in serum and cerebrospinal fluid were negative. His HbAc was 5.9% and blood glucose was 4.88 mmol/L. His anti-GAD antibody and anti-insulin antibody in serum was negative. Regular urinalysis and hearing was normal. The photographic images of the patient’s eyes showed bilateral papillary atrophy (Fig. 1). OCT measure around the disc showed thin retina (Fig. 2). Brain MRI showed bilateral optic nerve atrophy thinner (Fig. 3). OA and DM1 in a young patient suggested his clinically diagnose of WS.

The patient was the first child, and had one little sister. Both his little sister and his parents had no signs or symptoms. And his parents were non-consanguineous.

**Fig. 4:**
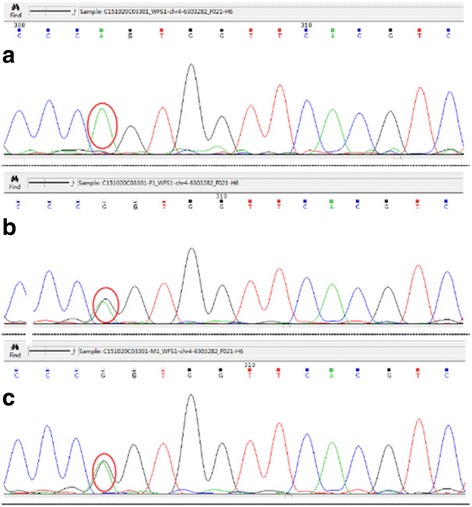
Exome sequencing. WFS1 exon 8 forward sequences of a homozygote for the patient (**a**), and heterozygotes for his father (**b**) and mother (**c**)

For further confirmation of the diagnosis, we got the consent to sequence WFS1 gene for the patient and his parents without his other family members. The patient’s number 587 codon, c.1760G > A, located in exon 8, mutated and changed from arginine to glutamine. He was homozygote while his parents were both heterozygote of mutant genes. We used the Human Gene Mutation Database (HGMD) to assess that this was a novel mutation. Exome sequencing disclosed a novel mutation in WFS1 gene (the novel variant c.1760G > A) (Fig. 4), confirming WS.

## Discussion and conclusions

WS is a rare progressive neurodegenerative hereditary disease, known as DI-DM-OA-D which stands for diabetes insipidus, diabetes mellitus, optic atrophy, and deafness [1]. The coexistence of T1DM and OA in juvenile suggests WS but molecular confirmation is mandatory [7]. Patients usually present with DM followed by OA in the first decade. DM is often the first clinical sign of WS, but differ from classical T1DM in normal autoimmune laboratory parameters. Patients with WS usually demonstrate progressive ophthalmologic symptoms. Deafness in WS is commonly a high frequency, symmetric hearing loss, usually detected in the second or third decade with a relatively slow rate of deterioration [8, 9]. Some studies have reported urological abnormalities which were expected about 58% in patients with WS [10].

Studies showed that WS was caused by loss-of-function mutations in the WFS1 gene, encoding wolframin [6]. Wolframin is abundantly expressed in pancreas, brain, heart, and muscle, with lesser amounts being present in liver and kidneys [11]. Although no function has yet been attributed to wolframin. Recently, it has been shown that WFS1 gene has a crucial role in the negative regulation of a feedback loop of the ER stress signaling network and preventing secretory cells. For example, wolframin deficiency in mice leads to progressive loss of B cells and impaired glucose tolerance [12]. Yamamoto H et al. concluded that dual dysfunction of wolframin in optic nerve glial cells and retinal ganglion cells in the cynomolgus monkey might explain the progressive optic nerve atrophy in WS [13].

The prognosis of WS is currently poor as most patients die at the age of 30s (range, 25–49 years) because of respiratory failure as a result of brain stem atrophy [1, 10].

But our case demonstrates an unusual presentation. The boy reported here onset with OA and T1DM, and both deafness and urological abnormalities were not found. His parents were non-consanguineous and were found mutant heterozygote while he was mutant homozygote. His condition was stable with a follow-up of 12 months.

Vision loss in Neurology was usually associated with ON or NMO. NMO can be associated with other autoimmune disease, such as T1DM [14]. So it is necessary to differentiate WS from NMO with T1DM in children.

Based on the findings of the present case, we should be aware of WS in adolescence patients presenting with T1DM (non-autoimmune) and OA without any signs of diabetic retinopathy. And it is mandatory to perform direct sequencing analysis of the WFS1 gene to confirm the clinical diagnosis. No genotype-phenotype correlation has been identified [6]. Although our study increases the spectrum of WFS1 gene mutations with a novel variant c.1760G > A, whether the novel mutation relates with mild clinical symptoms or just involves vision and blood glucose remains to be further studied and confirmed.

## Abbreviations

ANA: Anti-nuclear anti-body
ANCA: Anti-neutrophil cytoplasmic antibodies
AQP4: Aquaporin 4
D: Deafness
DI: Diabetes insipidus
DM: Diabetes mellitus
ENA: Extracted nuclear antigens
ER: Endoplasmic reticulum
HbAc: Glycosylated hemoglobin
HGMD: Human Gene Mutation Database
MRI: Magnetic resonance image
NMO: Neuromyelitis optica
OA: Optic atrophy
OCB: Oligoclonal bands
OCT: Optic coherence tomography
ON: Optic neuritis
TSH: Thyroid stimulating hormone
VEP: Visual evoked potential
WFS1: Wolfram syndrome 1
WS: Wolfram syndrome
